# Non‐invasive in vivo monitoring of transplanted stem cells in 3D‐bioprinted constructs using near‐infrared fluorescent imaging

**DOI:** 10.1002/btm2.10216

**Published:** 2021-03-26

**Authors:** Soon Hee Kim, Jin Seon Kwon, Jae Gu Cho, Kate G. Park, Tae Hyeon Lim, Moon Suk Kim, Hak Soo Choi, Chan Hum Park, Sang Jin Lee

**Affiliations:** ^1^ Wake Forest Institute for Regenerative Medicine, Wake Forest School of Medicine, Medical Center Boulevard Winston‐Salem North Carolina USA; ^2^ Nano‐Bio Regenerative Medical Institute, College of Medicine, Hallym University Chuncheon Republic of Korea; ^3^ Department of Molecular Science and Technology Ajou University Suwon Republic of Korea; ^4^ Department of Otolaryngology‐Head and Neck Surgery Korea University College of Medicine Seoul Republic of Korea; ^5^ Gordon Center for Medical Imaging, Department of Radiology Massachusetts General Hospital and Harvard Medical School Boston Massachusetts USA; ^6^ Department of Otorhinolaryngology‐Head and Neck Surgery Chuncheon Sacred Heart Hospital, School of Medicine, Hallym University Chuncheon Republic of Korea

**Keywords:** near‐infrared fluorescence, non‐invasive monitoring, scaffold monitoring, stem cell tracking

## Abstract

Cell‐based tissue engineering strategies have been widely established. However, the contributions of the transplanted cells within the tissue‐engineered scaffolds to the process of tissue regeneration remain poorly understood. Near‐infrared (NIR) fluorescence imaging systems have great potential to non‐invasively monitor the transplanted cell‐based tissue constructs. In this study, labeling mesenchymal stem cells (MSCs) using a lipophilic pentamethine indocyanine (CTNF127, emission at 700 nm) as a NIR fluorophore was optimized, and the CTNF127‐labeled MSCs (NIR‐MSCs) were printed embedding in gelatin methacryloyl bioink. The NIR‐MSCs‐loaded bioink showed excellent printability. In addition, NIR‐MSCs in the 3D constructs showed high cell viability and signal stability for an extended period in vitro. Finally, we were able to non‐invasively monitor the NIR‐MSCs in constructs after implantation in a rat calvarial bone defect model, and the transplanted cells contributed to tissue formation without specific staining. This NIR‐based imaging system for non‐invasive cell monitoring in vivo could play an active role in validating the cell fate in cell‐based tissue engineering applications.

## 1 INTRODUCTION

Tissue engineering involves the combination of cells with natural or synthetic biomaterials to create implantable tissue constructs, and it offers an attractive approach for the treatment of damaged tissues and organs. A large number of successful tissue engineering strategies require the use of mesenchymal stem cells (MSCs). MSCs hold great therapeutic promise as a cell source, and they can differentiate into multiple cell types, such as adipocytes, osteoblasts, and chondrocytes.^1^ The ability of MSCs to evade immunosurveillance after cell transplantation and to suppress the immune response has made MSCs attractive candidates for clinical use. While the therapeutic effects of stem cells are widely accepted,^2^ the specific mechanisms of tissue formation and the role of MSCs in the tissue‐engineered complex are poorly understood. To better understand the therapeutic effects of tissue‐engineered constructs after transplantation, monitoring of cell behaviors such as migration, survival and death, and differentiation in vivo should be performed. Recent advances in molecular imaging have allowed for the non‐invasive monitoring of transplanted cells,^3^, ^4^, ^5^ tissue formation,^6^, ^7^ and scaffold degradation^6^, ^8^, ^9^, ^10^, ^11^, ^12^, ^13^ in vivo without retrieving the implants. Non‐destructive and longitudinal consecutive monitoring provides more reliable information and is both more ethical and economical than invasive methods.

Bioprinting is a relatively novel technology that creates 3D structures consisting of biomaterials, living cells, and biomolecules. It has brought about a revolution in the field of tissue engineering, allowing for the fabrication of precise and sophisticated structures in a patient‐specific manner. Bioink is printable soft biomaterials loaded with living cells and is one of the main components of bioprinting technology. Bioink materials should be printable, degradable, non‐toxic, cytocompatible, and possess high mechanical properties. Hydrogels, which can be produced from proteins and extracellular matrix components (e.g., collagen and hyaluronic acid), provide environmental cues that directly aid stem cell growth. Hydrogels have been chosen as materials for 3D bioprinting due to their cell‐friendly and cross‐linkable properties.^14^, ^15^, ^16^ Hydrogel precursor solution is typically the state of a bioink during 3D printing. Crosslinking step is necessary to make a hydrogel network, and covalent crosslinking, unlike relatively weak physical crosslinking, increases strength and durability after 3D printing, and many studies have been conducted to impart covalent functionalization to natural hydrogels. Functionalized gelatin methacryloyl (GelMA) is relatively stable at body temperature, more resistant to degradation than gelatin, and at the same time retains the bioactive properties of gelatin such as cell attachment and enzymatic degradation.^16^, ^17^, ^18^, ^19^

Near‐infrared (NIR) fluorescence has advantages for biomedical imaging, including relatively low tissue absorption, reduced scattering, and minimal autofluorescence.^20^ Unlike visible light, which penetrates living tissue to a depth of less than a millimeter, NIR light can penetrate living tissue to a depth ranging between millimeters and centimeters. Thus, the use of NIR fluorophores produces a high signal that is detected with low background. In addition to imaging, there is a recent report that NIR has been used as a light source enabling non‐invasive 3D bioprinting due to its penetration into deep tissue and biocompatibility,^21^ and its application range is gradually expanding.

**FIGURE 1:**
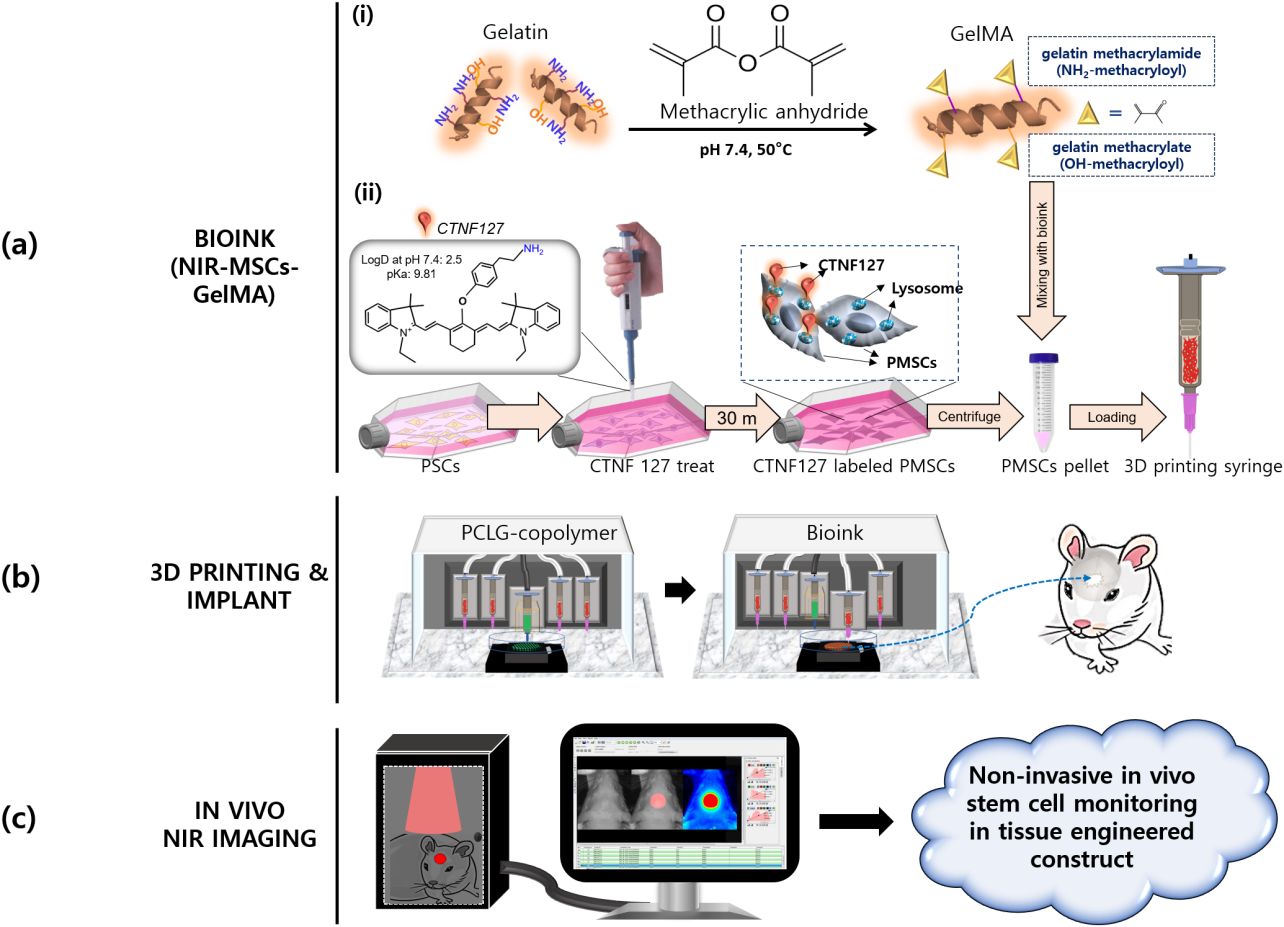
Schematic of the non‐invasive near‐infrared (NIR) imaging system used to track stem cells (a) Bioink prepared by mixing GelMA‐based materials (including other excipients) and NIR‐mesenchymal stem cells (MSCs). (i) GelMA synthesis, (ii) NIR‐MSCs preparation. NIR‐MSCs: Fluorescence‐emitting (700 nm) CTNF127‐labeled placenta‐derived mesenchymal stem cells (PMSCs) (b) 3D bioprinting of hybrid constructs; PCLG‐copolymer for a framework and NIR‐MSCs‐GelMA for cells were printed alternately. (c) Transplantation into rat calvarial defect and non‐invasive stem cell imaging

Previously, we reported that NIR fluorescence could be used to non‐invasively monitor scaffold degradation in vivo by employing zwitterionic NIR fluorophore (ZW800‐1) or highly charged cyanine^6^, ^22^ and cell growth in vitro using functional polymethine indocyanines.^23^ In this study, we tried to create a system that can be used to track cells embedded in a 3D bioprinted tissue‐engineered construct in vivo. GelMA bioink that impregnates NIR fluorophore‐labeled mesenchymal stem cells (NIR‐MSCs‐GelMA) was used as a bioink and analyzed signal stability and reliability after 3D bioprinting in vitro. Finally, we proposed that NIR‐MSCs‐GelMA could be an effective tool for non‐invasive tracking of transplanted cells in vivo and confirming the role of transplanted cells in tissue formation without specific staining (Figure 1).

## 2 RESULTS AND DISCUSSION

### 2.1 Intracellular trafficking of NIR fluorophores

We utilized placenta‐derived mesenchymal stem cells (PMSCs) because they are a promising cell source for regenerative medicine. PMSCs are obtained from the placenta, a tissue that is involved in multiple essential roles, including fetal development, tolerance, and serving as a reservoir of progenitor/stem cells. PMSCs have a remarkable ability to both differentiate into multiple cell types (encompassing the three germ layers), and to sustain undifferentiated proliferation for prolonged periods.^24^, ^25^ A high yield of PMSCs can be obtained from placentas discarded after childbirth. In addition, PMSCs expand quickly in vitro, produce a subdued immune response in recipients, and present enhanced immunosuppressive properties compared with bone marrow‐derived stem cells.

Several lipophilic organic fluorophores have been developed to label cytoplasm membrane, mitochondria, lysosomes, and so forth. Tagging membranes can interfere with cell migration and tagging mitochondria can affect membrane potential changes. On the other hand, tagging lysosomes as an intracellular digestive system minimizes the effect on cellular activity.^26^, ^27^ CTNF127 is a functionalized fluorophore that is selectively targeted to lysosomes and has excellent physicochemical and optical properties in serum. Furthermore, this dye is available at an NIR wavelength, and this enables the longitudinal tracking of living cells in vivo with minimal absorption, scattering, and autofluorescence.^27^, ^28^

**FIGURE 2:**
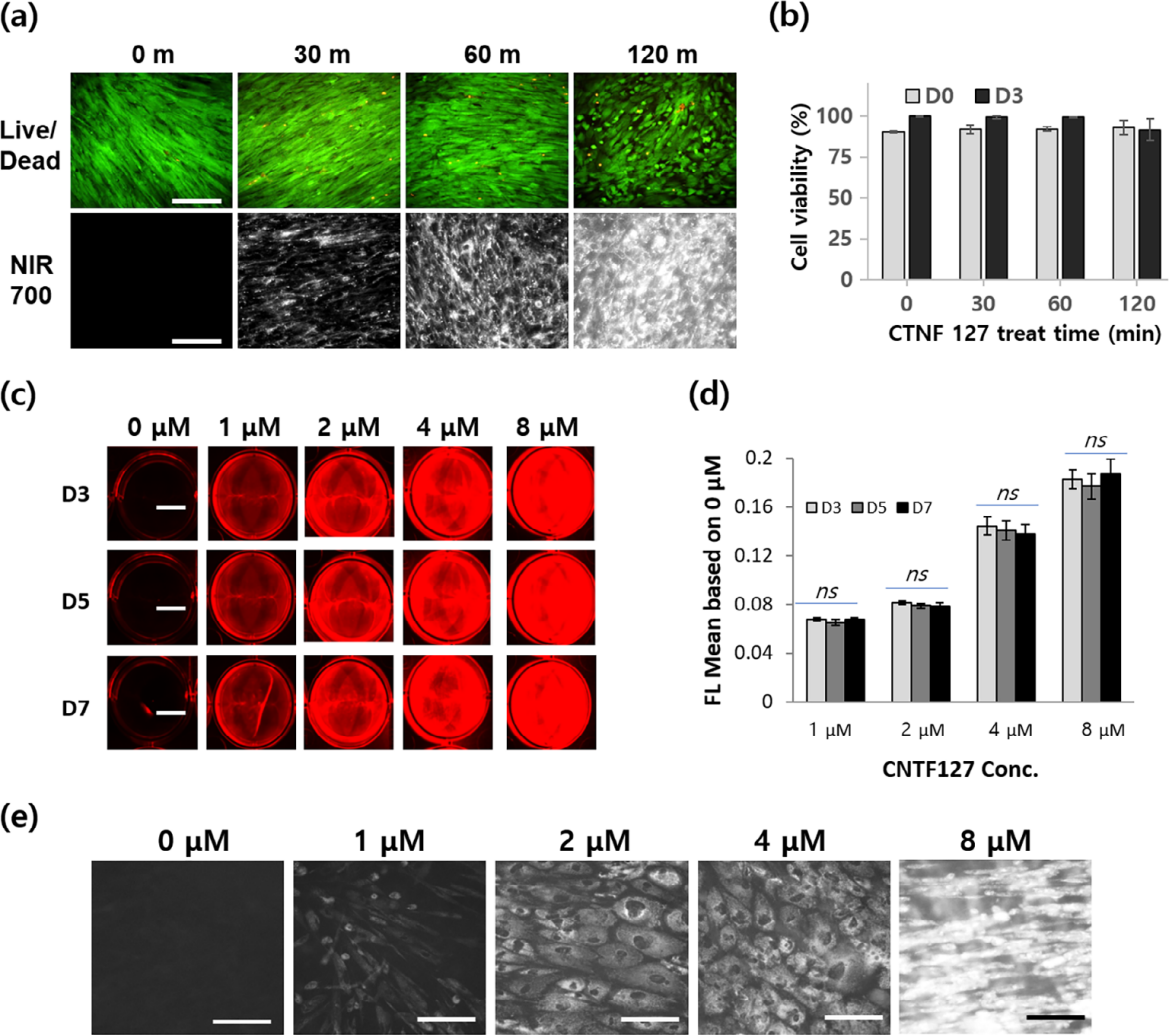
Optimization of labeling conditions (a, b) cytocompatibility using live/dead assay and fluorescence emission depending on CTNF127 (2 μM) labeling time at day 3 post‐cultivation and their quantification. Scale bars = 250 μm, (c, d) Stability and reliability of near‐infrared (NIR) fluorescence signal intensity as a function of fluorophore concentration (0–8 μM) and culture time (for 7 days), and their quantification. Scale bars = 1.5 cm, ns: not significant (*p* < 0.05 vs. D3). (e) Microscopic observation at day 20 post‐cultivation (exposure time: 2 s). Scale bars = 250 μm

Before the main experiments, we optimized the CTNF127 labeling of PMSCs (Figure 2). First, PMSCs were treated with NIR fluorophores (2 μM CTNF127) for various incubation times (30, 60, and 120 min) (Figure 2a). The live/dead assay revealed that most of the cells were viable when treated with CTNF127, regardless of incubation time. The fluorescent signal intensity increased as a function of incubation time. Using the MTS assay (cell viability), we demonstrated that the NIR fluorophores are cytocompatible (between 30 and 120 min of incubation) (Figure 2b). Next, PMSCs were treated with the NIR fluorophores at concentrations of 1, 2, 4, and 8 μM for 30 min. Table 1 shows the labeling ratio of CTNF127 to PMSCs. The increase in labeled dye amount was proportional to the CTNF127 concentration, but the labeling ratio increased with the initial treated amount up to 4 μM and then decreased at 8 μM. This suggested that a more concentrated fluorophore solution could diffuse more rapidly into the cytoplasmic membrane and react with acidic organelles in PMSCs easily (but there might be a critical point between 4 and 8 μM). Macro‐observations demonstrated that PMSCs had a strong NIR fluorescence intensity, and this intensity increased as the concentration of NIR fluorophores increased (Figure 2c). Additionally, the signal intensity was constant with time (day 3–7) regardless of the concentration used (1–8 μM) (Figure 2d). This finding suggests that CTNF127 was stably trapped inside lysosomes without cellular efflux. The micro‐observations (Figure 2e) demonstrate that PMSCs still expressed fluorescence on the culture dish 20 days post‐cultivation. The signal intensity gradually increased as a function of concentration, and the cell morphology looked healthy. These results demonstrate that CTNF127 is cytocompatible and stable in cells, which is important for providing reliable values when monitoring cell fluorescence. Based on these results, a concentration (2 μM) and exposure time (30 min) were selected as the optimal conditions and used for the rest of the study.

**TABLE 1:** Labeling ratio depending on CTNF127 concentration used to treat PMSCs

CTNF127 Conc. (μM)	Treated CTNF127 (nmole)	Labeled CTNF127 (nmole)	Labeling ratio (%)
1	1.03	0.42 ± 0.20	40.9
2	1.66	1.21 ± 0.10	72.9
4	3.41	2.61 ± 0.11	76.6
8	9.55	4.32 ± 0.03	45.2

Abbreviation: PMSCs, placenta‐derived mesenchymal stem cells.

### 2.2 Synthesis and characterization of GelMA

GelMA has natural cell‐binding motifs, hydrophilicity, and matrix metalloproteinase degradation sites.^29^ GelMA is water‐soluble, enhances cellular adhesion, and can degrade gradually in the body. The methacryloyl (MA) groups enable the GelMA to be light‐cured with UV treatment. GelMA has different physical properties (i.e., elastic modulus, water swelling, degradation, etc.) depending on the synthesis process; therefore, it is important to establish appropriate conditions according to the purpose of tissue engineering. In particular, the degree of methacryloyl substitution that directly affects the degree of crosslinking and pore size of hydrogel can be controlled by the amount of MA during synthesis.^30^, ^31^

We synthesized GelMA using various amounts of methacrylic anhydride. The GelMA solution before lyophilization was analyzed by ^1^H NMR that is often used to evaluate the replacement of free amino groups on gelatin by methacrylate groups (Figure S1). In the NMR peaks, the grafted methacryloyl group was verified using signals at *δ* = 5.4 ppm, *δ* = 5.7 ppm (acrylic protons, 2H), and by another peak at *δ* = 1.8 ppm (methyl group, 3H) (Figure S1a). In addition, the MA modification of lysine residues, according to the addition of methacrylic anhydride, can be confirmed by the decrease of the lysine signal at *δ* = 2.9 ppm (lysine methylene, 2H). The degree of substitution of methacrylamide on gelatin could be calculated using the lysine signal among NMR peaks.^32^ Consequently, it increased from 22% to 93% depending on the amount of methacrylic anhydride added (0.05–4 ml) (Table 2). We chose 0.6 ml of methacrylic anhydride based on stiffness of hydrogel fabricated with 5% GelMA (data not shown).

**TABLE 2:** The degree of methacrylate substitution according to methacrylic anhydride addition from ^1^H NMR analysis

Gelatin	Methacrylic anhydride (ml)	Average methacrylation (%)^a^
2 g (in 20 ml PBS)	4	93
	0.6	73
	0.1	45
	0.05	22
	0	0

Abbreviation: PBS, phosphate‐buffered saline.

^a^ The degree of methacrylate substitution for ^1^H NMR was determined by the formula: 1—(lysine integration signal of methacrylated‐substituted gelatin/lysine integration signal of unsubstituted gelatin).^31^

GelMA (hydrogels at concentrations (2.5% and 5%) were prepared to assess the gel stability depending on UV treatment time (Figure S1b). Nozzle clogging or pressure fluctuations were less in 5% GelMA than 2.5% GelMA (data not shown). As the GelMA concentration and UV treatment time increased, the opacity of the gel that was formed also increased, which indicated highly crosslinked gel formation. Furthermore, the formed gel maintained stable status after 15 days of incubation in PBS. For the rest study, 5% GelMA was mixed with the other components of bioink and crosslinked by UV for 90 s per one side following 3D printing. In other studies, 5% GelMA hydrogel showed high cell viability in vitro^33^ and excellent result for bone tissue regeneration.^34^ High crosslinking and high concentrations of polymer are more suitable to print structures with good shape fidelity and will be a hindrance to cell growth. More fine‐tuning is required in order to find the optimal biofabrication window.^35^

### 2.3 3D bioprinting using bioink NIR‐MSCs‐GelMA

**FIGURE 3:**
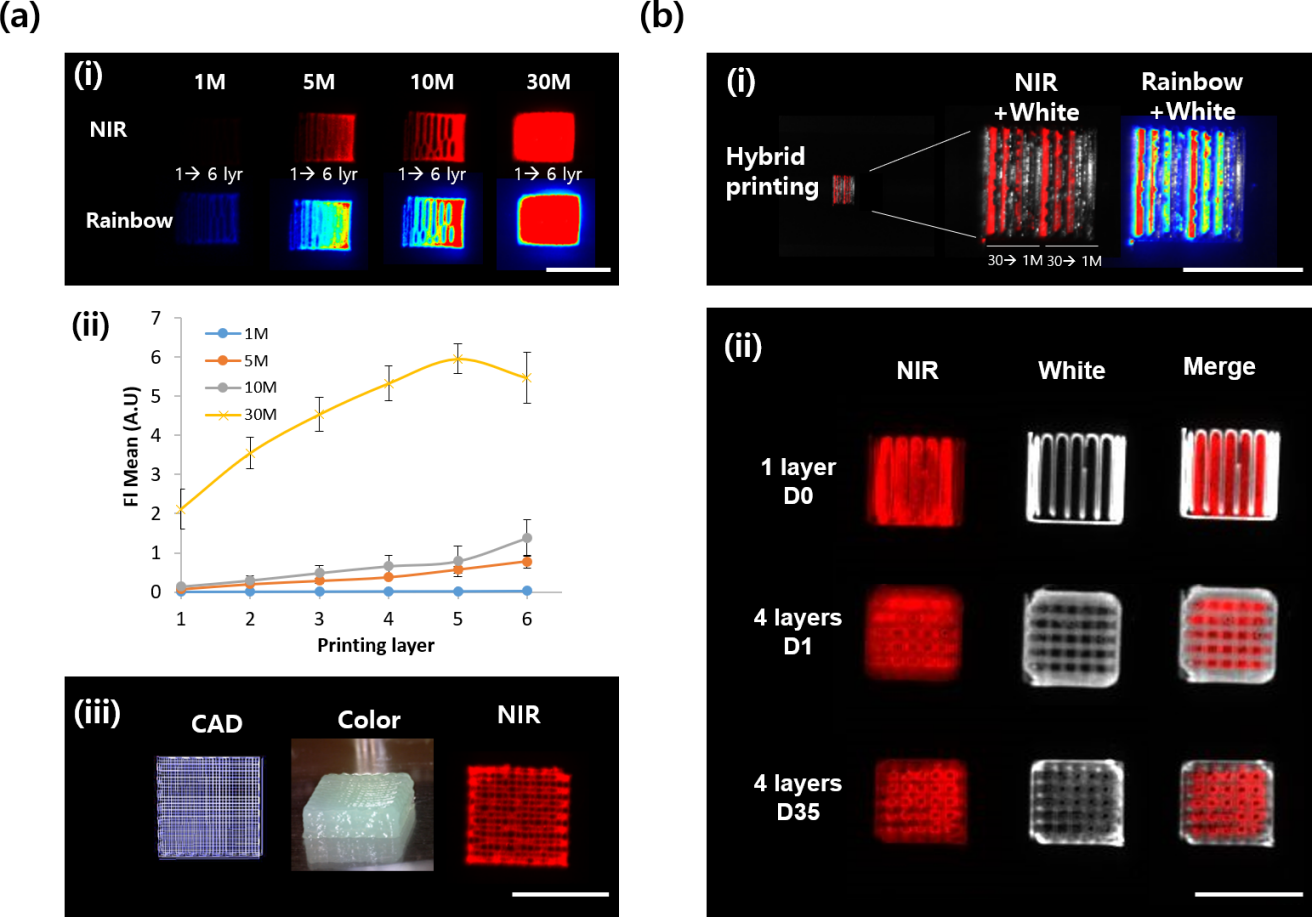
Optimization of printing condition using bioink, near‐infrared‐mesenchymal stem cells‐gelatin methacryloyl (NIR‐MSCs‐GelMA) (a) Single bioink printing. (i–ii) Fluorescence signal intensity from hydrogel printed by bioink with various cell numbers (1–30 M). Every strand in the same construct has different layer numbers (1–6). M: 1 × 10^6^ cells/ml. Fluorescence signal intensity was quantitatively increased by both the printing layer and cell number. The mean fluorescence was normalized by the fluorescence strength of GelMA containing unlabeled PMSCs. (iii) The bioink, composed of 10 M of NIR‐MSCs, was printed in 10 layers, and its imaging reveals sophisticated printing results that show the printed traces in macro‐fluoroscopic measurement. (b) Hybrid 3D bioprinting of bioink and PCLG‐copolymer (i) bioink with various cell numbers every strand (1–30 M) was used to print the alternate lines along PCLG‐copolymer, and their imaging was performed in a one‐layer structure. Bioink demonstrated a reliable increment of fluorescence intensity by cell number. The redder the color, the stronger the intensity in the rainbow image. (ii) Hybrid 3D bioprinting of bioink and PCLG‐copolymer with a lattice structure. NIR fluorescence (700 nm) was distinctly distinguished from the framework of construct regardless of layer number and culture time. Scale bars = 1 cm

The NIR‐MSCs‐GelMA was fabricated by mixing the GelMA‐based bioink and the NIR‐MSCs. NIR‐MSCs‐GelMA was printed in various conditions to validate its printability and fluorescence emission capability after printing (Figure 3). Initially, hydrogels with varying cell numbers were printed out in one structure with gradient printing layers (1–6 layers) (Figure 3a). The NIR fluorescence intensity was proportional to the cell number and printing layer in both single‐color and rainbow images (Figure 3a‐i). The signal intensity was proportional to cell number up to a seeding number of 10 million (10 M) cells, but at 30 M cells, this intensity was 2×–5× of the intensity at 10 M cells (Figure 3a‐ii). Our printing setup outputs a 10‐layer mesh‐type hydrogel using NIR bio‐ink containing 10 M cells. We carried out macroscopic observations of fluorescence and were able to see NIR fluorescence images showing finely printed traces from the structure (Figure 3a‐iii).

Next, we developed a bioink (NIR‐MSCs‐GelMA)‐incorporated PCLG‐copolymer using the 3D printer (Figure 3b). NIR‐MSCs‐GelMA was placed between the strands of the PCLG‐copolymer. Models of the complex showed that the fluorescence signal from each strand increased with the number of incorporated cells (Figure 3b‐i). When the NIR‐MSCs‐GelMA and PCLG‐copolymer were printed together increasing layers in lattice form, the bioink showed stable printability in the presence of the PCLG‐copolymer. Consequently, the NIR fluorescence signal (pseudo‐red) from the complex was also obtained at 700 nm. Furthermore, macro‐observation demonstrated that the NIR fluorescence was emitted from the same place in the complex even after 35 days of long‐term culture (Figure 3b‐ii).

**FIGURE 4:**
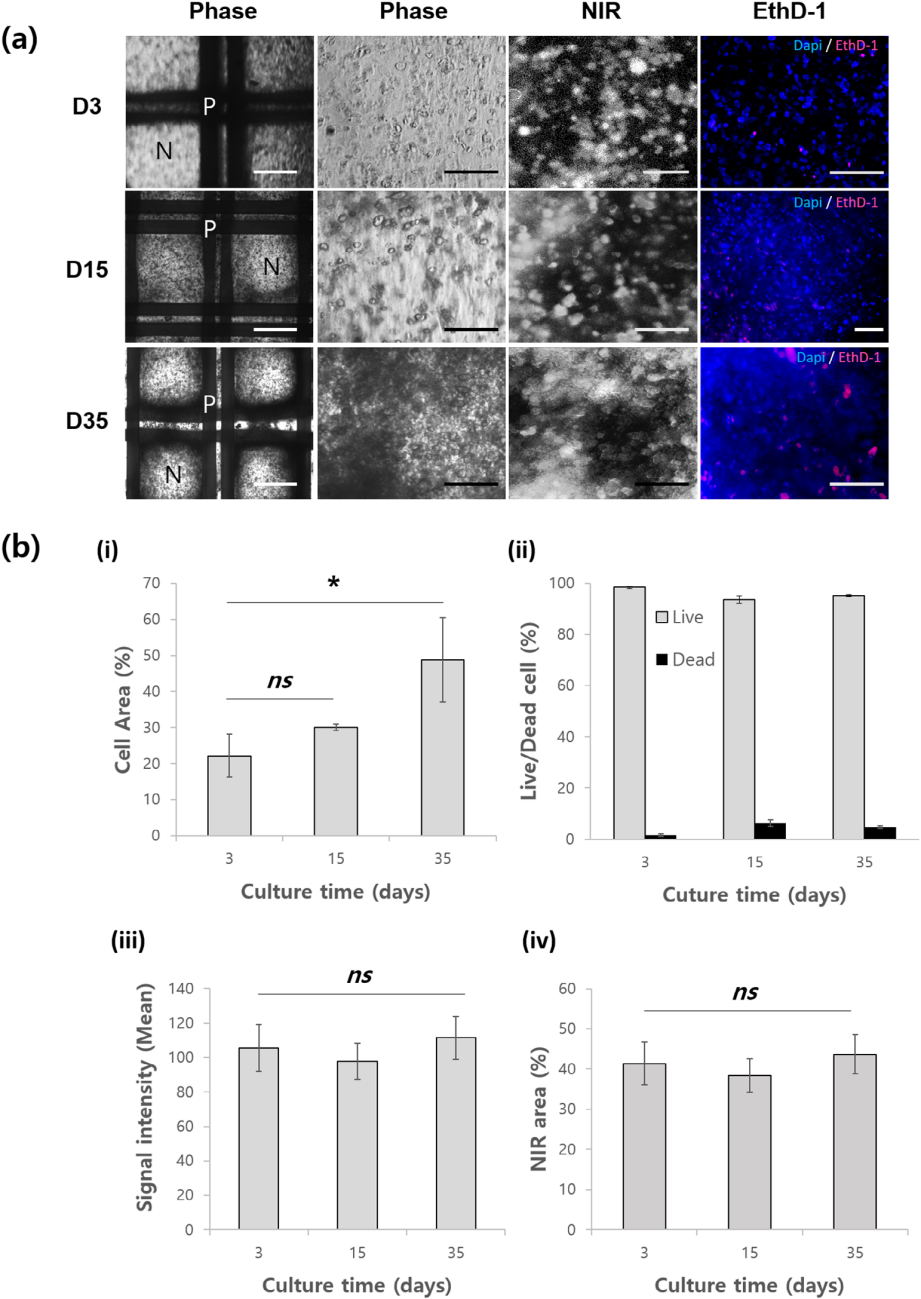
Long‐term cell viability and fluorescence maintenance in the complex. (a) Representative images from three culture time. Near‐infrared‐mesenchymal stem cells (NIR‐MSCs) shows high cell viability through Dapi/EthD‐1 staining and stable NIR fluorescence emission in long‐term culture (35 days) of the 3D printed complex. Scale bars = 1 mm for phase images, 250 μm for the rest of the images. P: PCLG‐copolymer, N: NIR‐MSCs‐GelMA printed area. Dapi: nuclei, EthD‐1: dead cells (b) Quantitative analysis of (a). (i) The overall number of cells increased in vitro. (ii) The number of living cells dominated the number of dead cells in the GelMA. The entire (iii) signal intensity and (iv) the signal area were not significantly different for 35 days. This reflects that the system composed of CTNF127‐labeled cells embedded in GelMA has high cell viability and low photobleaching and fluorescent efflux. *p < 0.05 versus day 3, ns: not significant

In long‐term microscopic observations, the fluorescence was shown to come from NIR‐MSCs incorporated within the bioink printed in the hybrid construct (Figure 4). The NIR‐MSCs in the complex showed active proliferation with normal morphology and emitted a strong NIR fluorescence signal at 700 nm in region of interest (ROI) (Figure 4a). Additionally, a few dead cells were detected within the constructs using EthD‐1 staining. These were shown quantitatively in Figure 4b‐i and ii. The total cell area increased with the incubation time, and the percentage of living cells over the entire period accounted for about 95.7% of the total area. In terms of cell proliferation, other studies also investigated that the use of GelMA in the 3D bioprinting to fabricate cell‐laden constructs showed high cell viability (77%–97%).^36^, ^37^ Our study demonstrates additional information that GelMA does not hinder to fluorescence signal emission from NIR fluorophore‐labeled cells. In quantitative analysis (Figure 4b‐iii and iv), the entire NIR fluorescence signal intensity and its signal area in the ROI was significantly invariable during test. Unlike indirect labeling using such as cell transfection with fluorescent proteins (e.g., green fluorescent protein), many direct labeling technologies commonly undergo halving of dye following cell division.^38^ Our result disproves that the fluorescence leakage from cells was minimized, which is expected to be due to the labeling effect of CTNF127 on the lysosomes of cells.^27^ These results demonstrate that CTNF127 can be a reliable fluorophore for use in 3D culture environments and would be suitable for long‐term cell imaging in 3D construct.

### 2.4 Non‐invasive monitoring of cells implanted in the tissue‐engineered construct in vivo

**FIGURE 5:**
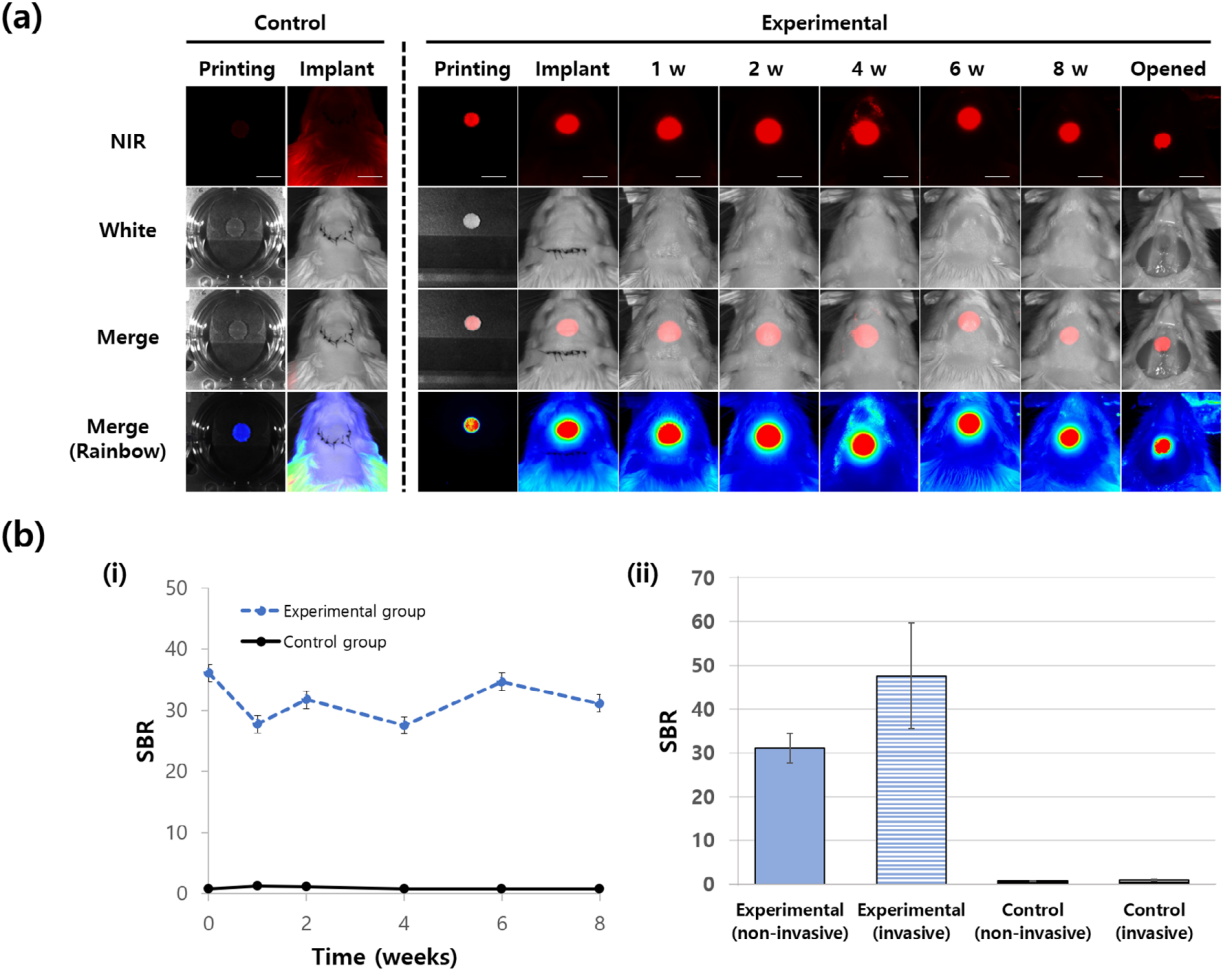
Non‐invasive in vivo stem cell monitoring. (a) Implantation of the construct into the rat calvarial defect site and the non‐invasive monitoring for 8 weeks. (i) Observation of experimental group implanted the construct with near‐infrared‐mesenchymal stem cells (NIR‐MSCs). NIR fluorescence was emitted from the implants at 700 nm. (The fluorescence at 4 weeks came from the unshaved fine hairs.) *Control group: the group implanted the constructs without NIR‐MSCs (see Figure S2 for extended analysis). Scale bars = 1 cm. Scale bars = 1 cm. (b) Quantitative analysis of (a) (i) SBR (signal to background) remained high and constant during monitoring. There was no significant difference between SBR immediately after implantation and each detection time (*p* < 0.05). (ii) Comparison of SBR via non‐invasive imaging (closed skin) and invasive imaging (open skin) at 8 weeks post‐surgery. The SBRs between them were not significantly different (*p* < 0.05)

Following successful in vitro results measuring the NIR fluorescence signal from NIR‐MSCs‐GelMA, the circular mesh‐type complexes were implanted into the rat calvarial defect site, and the fluorescence signal was monitored (over the skin) using dual NIR imaging apparatus (Figure 5). Previous other studies have shown that the NIR beam penetrates the periosteum (0.1 mm)^39^ and skin (1.2 mm).^40^ Even though NIR reduces autofluorescence from several organs including skin, gallbladder, and bladder,^20^ furs are not free from the autofluorescence at 700 nm. Therefore, we removed rat furs before performing in vivo imaging. The control group using rats implanted a construct without labeled cells did not show signals at 700 nm from the hair removed skin on the site where the construct was implanted (Figures 5a and S2). In the experimental group (implantation of the construct with NIR‐MSCs), we observed fluorescence signals from the cells within the implant at 700 nm wavelength. The fluorescence signal at 700 nm had a similar intensity and size when compared with the start of the experiment (Figure 5b‐i), indicating the preservation of implanted cells. This phenomenon was observed in the merged rainbow images and demonstrated that PMSCs stably exists in the hybrid construct during implantation. The substrate stiffness affects cell migration, which is important for cell migration from the scaffold toward host tissue and for the ingrowth of cells from host tissue.^41^ PMSCs stability in the scaffold might be affected by the high concentration of GelMA^42^ and long degradation time (half‐life >4 weeks) of PCLG copolymer.^43^

There was no significant difference in signal to background ratio (SBR) values for closed and open skin at 8 weeks post‐surgery (Figure 5b‐ii). This means that the fluorescence signal over the skin stably reflects the signal of the actual implants, which demonstrates the reliability of our imaging system. In previous studies, when the NIR fluorophore (ZW800)‐conjugated scaffold was implanted under the skin of a nude mouse and its degradation behavior was analyzed by non‐invasive fluorescence imaging, it was consistent with the invasive analysis results.^6^

### 2.5 Stem cell tracking by histological analysis

**FIGURE 6:**
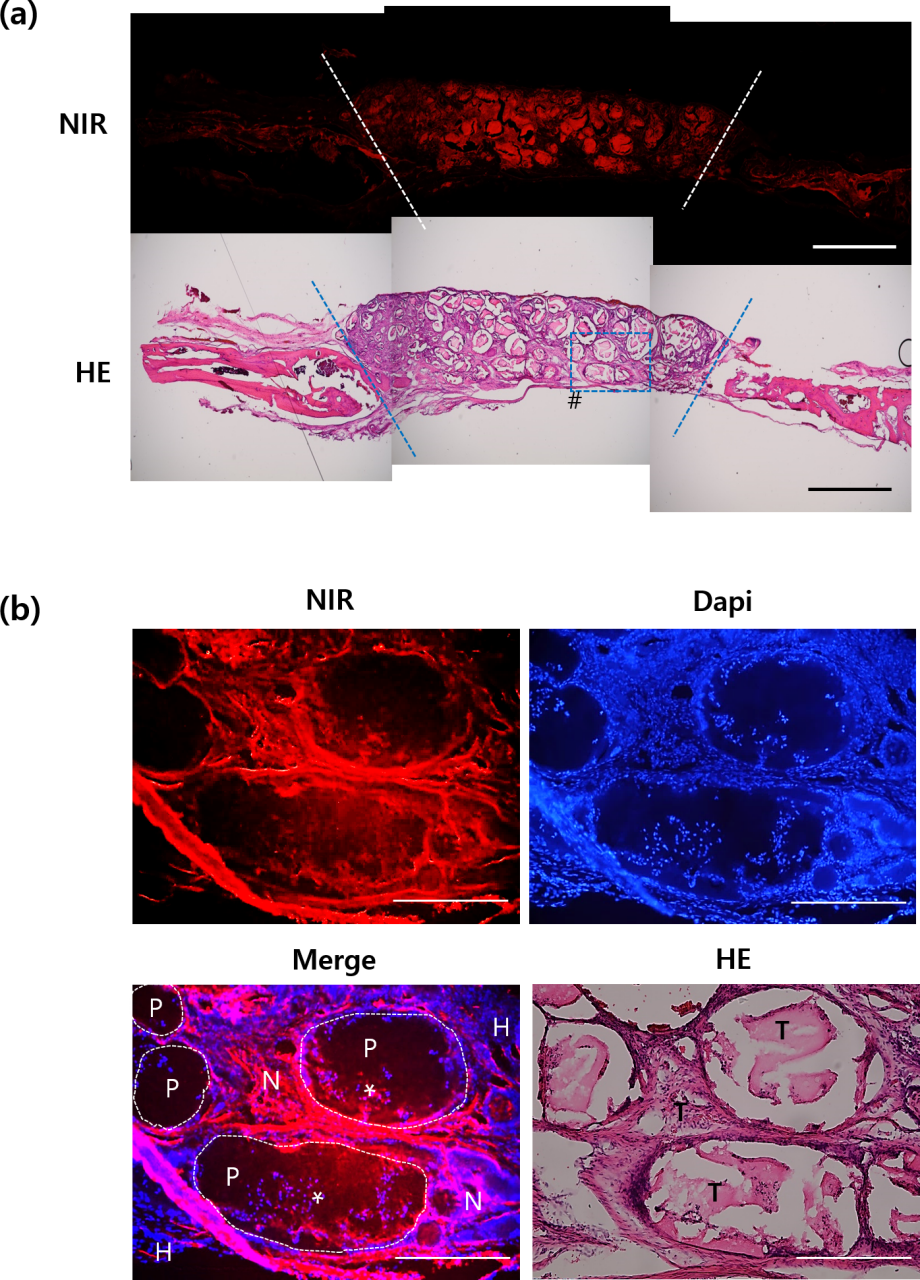
Stem cell tracking via comparison of near‐infrared (NIR) fluorescence and histological analysis of constructs implanted in calvarial defects for 8 weeks (a) total implanted area. Dotted lines mark the boundary of implant and host tissue. (b) Magnified images of dotted box (denoted as #) in (a). The dotted circle refers to the PCLG‐copolymer (denoted as P), and the surrounding area is the area printed with NIR‐MSCs‐GelMA. The red color indicates fluorescence from NIR‐MSCs (marked as N) and blue color from cells' nuclei within the area. H indicates host tissue. NIR‐MSCs infiltrated into PCLG‐copolymer were marked with an asterisk (*). It was found that the newly formed tissue area (denoted as T) on the HE stained slide overlaps the NIR‐MSCs region. Scale bars = 2.5 mm for (a) and 500 μm for (b)

The contribution of PMSCs to tissue formation is shown in Figure 6. At 8 weeks post‐transplantation, the complex was extracted and cryo‐sectioned (Figure 6a). An NIR fluorescence signal from the natural‐sectioned slide, without any particular histological staining, was observed at 700 nm. Hematoxylin and eosin (H&E) staining in the sequentially sectioned slide demonstrated tissue formation between scaffold materials, as standard staining procedures dissolved the common synthetic polymer. The relationship between tissue formation and NIR fluorescence signal reflected the position of stem cells and is shown in the magnified images (Figure 6b). Dark, rounded areas in the fluorescence image indicate portions of the PCLG‐copolymer that were initially printed. The pseudo‐red color at 700 nm surrounded the copolymer and indicated NIR‐MSCs. On the other hand, cells from host tissue did not show red signal (denoted as H). Interestingly, the red signal was overlapped in the site where the copolymer was located, and this part was also overlapped with the newly formed matrix when compared to the result of HE staining. This may be the result of the migration of NIR‐MSCs to the copolymer part following scaffold degradation. DAPI staining revealed that the region where the fluorescence signal was expressed at 700 nm was from implanted cells.

The main two modes of stem cell therapy are a direct replacement of dead cells and indirect healing through the release of paracrine factors.^44^ In order for stem cells to work in any way, understanding and monitoring the fate and regenerative potential of transplanted stem cells is essential to expediting the clinical application of stem cells by improving the safety and therapeutic efficacy of stem cell‐based therapies.^45^, ^46^ To accurately determine the degree of utilization of stem cells placed into the scaffold, a deeper study is required to detect the bio‐distribution, cell survival, and fate at the whole organism level. Nevertheless, the current study shows that the MSC encapsulated in the scaffold has achieved successful colonization and tissue formation, more importantly, our imaging system contributed to identifying this.

Preclinically, the effect of PMSCs have been proved in the various bone diseases such as osteogenesis imperfecta,^47^ myeloma bone disease and tumor growth,^48^ and in tissue engineering field. It has been reported that hPMSCs could facilitate the repair of bone defects by secreting osteogenically active factors.^39^, ^49^ PMSCs have been examined for bone tissue engineering using various biomaterials and showing encouraging results,^50^, ^51^ especially, in the calvarial defects of rats PMSCs seeded in PLGA porous scaffolds had the higher xenogenic reconstruction potential.^52^ Other studies using various MSCs, besides PMSCs, have been reported that MSCs contributed to bone reconstruction in the calvarial defects of mice, dogs, and rabbits.^53^, ^54^, ^55^, ^56^ We also expect that the newly formed tissue is bone extracellular matrix, however, the detail tissue characterization should be performed. Injecting additional fluorophores targeting specific tissues such as bone and cartilage^57^, ^58^ or targeting specific molecules such as fibrin^59^ and cathepsin B^60^ will be helpful for characterizing tissue formation or responses.

Although it can vary depending on the type and site of the injury, it was reported that the average bone healing time is between 6 and 8 weeks.^61^ In rodents, the calvarial defects can be filled by soft fibrous tissue suggesting the critical period of restoring bone is between 4 and 8 weeks.^62^, ^63^ As such, the 8‐week period is clinically relevant but is also a remarkable period for in vivo cell tracking studies. This period is longer than previous in vivo studies with labeled stem cells, including IR‐780 iodide‐labeled multipotent stem cells derived from rat skin dermis in rats (1 week),^64^ PKH26 fluorescent‐labeled human bone marrow‐derived MSCs transplanted in nude mice (4 weeks),^65^ and ultrasmall superparamagnetic iron oxide‐labeled human‐derived stem cells in nude mice (4 weeks).^5^ Even though studies that use labels that are bound with DNA (such as BrdU)^66^ or membrane proteins (such as GFP)^67^ have tracked cells for similar or longer timeframes (8–12 weeks), these compounds limit clinical translation.

## 3 CONCLUSIONS

Non‐invasive monitoring of cells in tissue‐engineered constructs is important to understand their therapeutic contribution post‐implantation. We demonstrated that NIR fluorophore‐labeled cells in the bioprinted constructs could retain fluorescence signals for long periods and were critical for non‐invasive in vivo monitoring using the NIR imaging system. Stem cells that have paracrine effects and the ability for direct differentiation are necessary for tissue restoration. Additionally, biomaterials are also necessary for improving the therapeutic effectiveness and successful delivery of stem cells. The results in this study emphasize the importance of the interrelationship between the scaffold and stem cells in this tissue engineering approach that incorporates the NIR non‐invasive imaging method. This approach could provide a more precise understanding of stem cell contribution during tissue regeneration. Furthermore, it may also address the challenges of using allogeneic stem cells for promoting tissue formation. Further studies focusing on the identification of newly formed tissue would verify the specific role of stem cells in the tissue‐engineered construct.

## 4 EXPERIMENTAL SECTION

### 4.1 Cultivation of PMSCs

Consent for acquiring the human placenta was obtained from mothers before giving birth and the fresh placenta was transported to Wake Forest Institute for Regenerative Medicine for tissue isolation.^68^ Briefly, fetal cells were isolated from chorionic placental tissue cells at full term. For placental cells, the whole placenta was collected, and a biopsy isolating the chorion was digested for fetal placental cells.^38^ We received a donation of quality‐controlled human placenta‐derived stem cells (PMSCs, passages 4–13) from the institution. PMSCs were cultured in α‐Minimum Essential Medium (α‐MEM) supplemented with 15% fetal bovine serum (FBS, HyClone, Logan, UT, United States), 17% AmnioMAX™ C‐100 Basal Medium, 2% AmnioMAX™ C‐100 Supplement, 1% GlutaMAX™ Supplement, and 2.5 μg/ml gentamicin. The medium was changed every 3 days. All reagents for cell culture were purchased from Life Technologies (Carlsbad, CA, United States) unless stated otherwise.

### 4.2 Cellular labeling with NIR fluorophore

The 700 nm NIR fluorescence‐emitting fluorophore (CTNF127) was synthesized based on the previous method.^27^ Cultured PMSCs were washed with phosphate‐buffered saline (PBS, pH 7.4) twice to remove serum, treated with 2 μM of CTNF127 (diluted in α‐MEM without supplements), and incubated (37°C, 5% CO_2_). After 30 min, PMSCs were washed with PBS thrice to remove unlabeled CTNF127. We referred to CTNF127‐labeled PMSCs as NIR‐MSCs for convenience. NIR‐MSCs were detached with 0.25% trypsin containing 1 mM EDTA for 5 min at 37°C.

### 4.3 GelMA‐based bioink loaded with CTNF127‐labeled MSCs (NIR‐MSCs‐GelMA)

Methacrylated gelatin was synthesized as described previously^69^ and used as the main bioink component. Briefly, 2 g of type A porcine skin gelatin (~300 g bloom) was fully dissolved in 20 ml of 10% (wt/vol) Dulbecco's PBS (DPBS) at 60°C. Methacrylic anhydride (0.6 ml) was added dropwise (0.5 ml/h) to the gelatin solution under stirring conditions at 50°C and allowed to react for 1 h. The GelMA solution (20 ml) was added to 80 ml of PBS (1:5 dilution). Next, the solution was dialyzed against deionized water (DW) using 12–14 kDa cutoff dialysis tubing (Spectra/Por®, Spectrum Chemical Mfg. Corp., Gardena, CA, United States) for 1 week at 40°C to remove salts and unreacted methacrylic acid. The water was changed twice daily. Finally, the GelMA solution was lyophilized for 1 week and stored at −80°C until further use. The degree of methacrylation was calculated by NMR spectroscopy. The bioink material for 3D bioprinting was completed by mixing 50 mg/ml GelMA macromer, 3.75 mg/ml hyaluronic acid, 12.5% (vol/vol) glycerol, 37.5 mg/ml of gelatin (bloom 90–110), and 0.2% (wt/vol) Irgacure 2959 as a photoinitiator in α‐MEM medium. This material was incubated at 40°C until fully dissolved, filtered using a 0.45 μm syringe filter, and mixed with NIR‐MSCs at a concentration of 1 × 10^7^ cells/ml. The bioink laden with NIR‐MSCs is referred to as NIR‐MSCs‐GelMA in this study.

### 4.4 3D bioprinting using NIR‐MSCs‐GelMA

The 3D bioprinter (Integrated Tissue‐Organ Printer, ITOP) and CAD software, including slicing, tool path generation, and motion program generation, used in this study were developed in‐house.^70^ The 3D bioprinter is composed of six cartridges that enable the printing of various materials. NIR‐MSCs‐GelMA with different cell numbers (0.1–3 × 10^7^ cells/ml) were loaded into four plastic syringes fitted with a 300 μm Teflon nozzle for 3D bioprinting. Bioprinting was carried out at 150 kPa air pressure. After printing, the upper and lower sides of the printed 3D structures were each crosslinked by UV light with a 320–500 nm filter (Exfo Omnicure S1000 lamp, Excelitas Technologies, ON, Canada) at a power density of 10 mW∙cm^−2^ for 90 s at room temperature.

### 4.5 Hybrid 3D bioprinting using NIR‐MSCs‐GelMA and PCLG‐copolymer

For a framework of constructs, mPEG‐(PCL‐ran‐PLLA‐ran‐PGA) (PCLG, CL32/LA58/GA10) copolymer was synthesized according to protocols derived from previous studies.^6^, ^71^ A metal syringe in a 3D printer was loaded with PCLG‐copolymer and heated to 105°C for melting. One of the plastic syringes was loaded with NIR‐MSCs‐GelMA (plus 1 × 10^7^ cells/ml). The molten PCLG‐copolymer was extruded through a 300 μm conical metal nozzle at 500 kPa to form a lattice, which was deposited in a layer‐by‐layer fashion at a velocity of 170 mm/min. Simultaneously, NIR‐MSCs‐GelMA was alternately printed at every layer with the conditions mentioned above Section 4.4. A single‐strand of NIR‐MSCs‐GelMA was equivalent to the height of two‐stranded PCLG‐copolymer. The temperature in the chamber was maintained at 22°C during the printing process. For the in vitro study, the size of the hybrid construct was 8 × 8 mm and ~2.4 mm thick. For the in vivo study, round‐shape scaffolds of 7 mm diameter and 1 mm thickness were printed out. The hybrid construct was printed with a gradient pore size (top: 300 μm pitch to bottom: 600 μm pitch) to minimize host cell infiltration.^70^ After printing, the hybrid 3D constructs were UV‐crosslinked (Section 4.4).

### 4.6 Cell viability in the NIR‐construct

The relationship of cytotoxicity to the CTNF127 treatment time was evaluated using a Live/Dead assay kit (Life Technologies, United States), according to the manufacturer's instructions. Cell viability in the hybrid construct was analyzed with DAPI staining and ethidium homodimer (EthD‐1) staining to observe dead cells at day 15 and 35. Cell proliferation was examined using the MTS assay kit (Abcam, Cambridge, United Kingdom) according to the manufacturer's protocol, and determined by absorbance at 490 nm.

### 4.7 NIR‐construct implantation

Animal studies were performed under the supervision of the Wake Forest University of Medicine Institutional Animal Care and Use Committee (IACUC) in accordance with an approved institutional protocol #A15‐051. The calvarial bone defect model was created in six Sprague Dawley rats (male, 8 weeks old, Charles River Laboratory). Rats were anesthetized using 3% isoflurane (before the surgical procedure) and maintained with 2% isoflurane throughout all surgical procedures. During the procedure, the animal was positioned on a warming pad. The hair over the skull was shaved, and the underlying skin was aseptically prepared using povidone‐iodine/betadine scrub. The skin was roundly incised, and the subcutaneous tissue was dissected along the same line as the skin. The underlying periosteum was sharply incised and subsequently elevated off the skull to obtain sufficient exposure for defect creation. A cooled stainless‐steel trephine (7 mm diameter) and a surgical drill were used to remove a full‐thickness section of bone. The 3D‐printed complex was implanted after separating the bone flap from the dura mater underneath with a Malis dissector. Meticulous hemostasis was maintained throughout the procedure via epinephrine gauze and manual compression. The implants were secured by snug placement and by the closure of the overlying fascia. For non‐invasive fluorescent imaging, the periosteum and subcutaneous tissue were closed with 5–0 absorbable suture, and the skin was closed with 4–0 non‐absorbable suture in an interrupted pattern. The suture was removed 3 days after surgery. Animals were hosted in the WFIRM animal care center.

### 4.8 NIR fluorescence imaging

Micro‐fluorescence images were obtained using a fluorescence microscope (TE2000‐U, Nikon, Melville, NY, United States) equipped with a 175 W full‐spectrum Xenon lamp, QuantEM EMCCD camera (512SC, Photometrics, Tucson, AZ, United States), NIR‐compatible optics (CY5.5 and CY7 Filter Set), an NIR‐compatible 10× Plan Fluor objective lens (Nikon), and DAPI filter set. Images were converted to pseudocolour (red/green) and analyzed by InnerView™ (INNERVIEW Co., Sungnam, Korea).

Macro‐fluorescence images were taken using a small animal in vivo imaging system (Pearl® Impulse, LI‐COR Biosciences, Lincoln, NE, United States), which was equipped with NIR channels (700 and 800 nm) and a white channel. The intensity of the NIR fluorescence signal from rats was monitored over the skin weekly for 8 weeks, while the rats were anesthetized with 3% isoflurane. In this study, the imaging system was remodeled to be used with rats (to avoid size limitations). Rats were sacrificed at 8 weeks for imaging after skin removal and histological analysis. Imaging data were collected and quantified by Pearl Software Images (LI‐COR Biosciences). The fluorescence region seen in the earliest sample was determined by the ROI. The mean of the fluorescence intensity (FL Mean; arbitrary unit) was presented in the results.

### 4.9 Histological analysis

Transplanted sites including implants from rats were harvested and decalcified in Richard Allan Scientific Decalcifying Solution (Thermo Scientific) for 24 h following fixation with 10% neutral‐buffered formalin for 48 h. Decalcified tissues were rinsed with DW, dipped in 30% sucrose for 1 day, and frozen in liquid nitrogen for cryo‐embedding. Frozen samples were sequentially cryo‐sectioned (10 μm per slice). One of the slides was used to observe the NIR fluorescence signal at 700 nm. Another slide was used to assess DAPI staining (nuclei). To visualize tissue formation, the other sectioned slide was stained with H&E after fixation with 4% paraformaldehyde for 10 min. All the images were obtained using the NIR fluorescence microscope described above Section 4.8.

### 4.10 Statistical analysis

The samples were assessed in triplicate for each group to analyze the statistical data. The data are presented as the mean value ± SD. Statistical analysis of the experimental results was performed using one‐way analysis of variance followed by the Tukey multiple comparisons test. The reported *p* values were considered statistically significant at *p* < 0.05.

## AUTHOR CONTRIBUTIONS

**Soon Hee Kim:** Conceptualization; data curation; formal analysis; investigation; methodology; software; visualization; writing‐original draft; writing‐review & editing. **Jinseon Kwon:** Investigation; methodology. **Jae Gu Cho:** Investigation; methodology. **Kate G. Park:** Investigation. **Tae Hyeon Lim:** Visualization. **Moon Suk Kim:** Conceptualization; data curation; formal analysis; resources; supervision. **Hak Soo Choi:** Conceptualization; data curation; formal analysis; resources; supervision. **Sang Jin Lee:** Conceptualization; formal analysis; funding acquisition; project administration; resources; supervision; writing‐review & editing.

## CONFLICT OF INTEREST

Authors declare no conflicts of interest.

## Supporting information


**Appendix S1**: Supporting Information

## Data Availability

Data available in article supplementary material
