# Supplementary material for: Non‐invasive in vivo monitoring of transplanted stem cells in 3D‐bioprinted constructs using near‐infrared fluorescent imaging
Source: Bioeng Transl Med. 2021 Mar 26;6(2):e10216. doi: 10.1002/btm2.10216 (PMC8126817; doi:10.1002/btm2.10216)
Supplement: Supplementary file 1 — Appendix S1: Supporting Information [file BTM2-6-e10216-s001.docx]

**Supporting Information**

**Non-invasive *in vivo* monitoring of transplanted stem cells in 3D-bioprinted constructs using near-infrared (NIR) fluorescent imaging**

*Kim et al.*


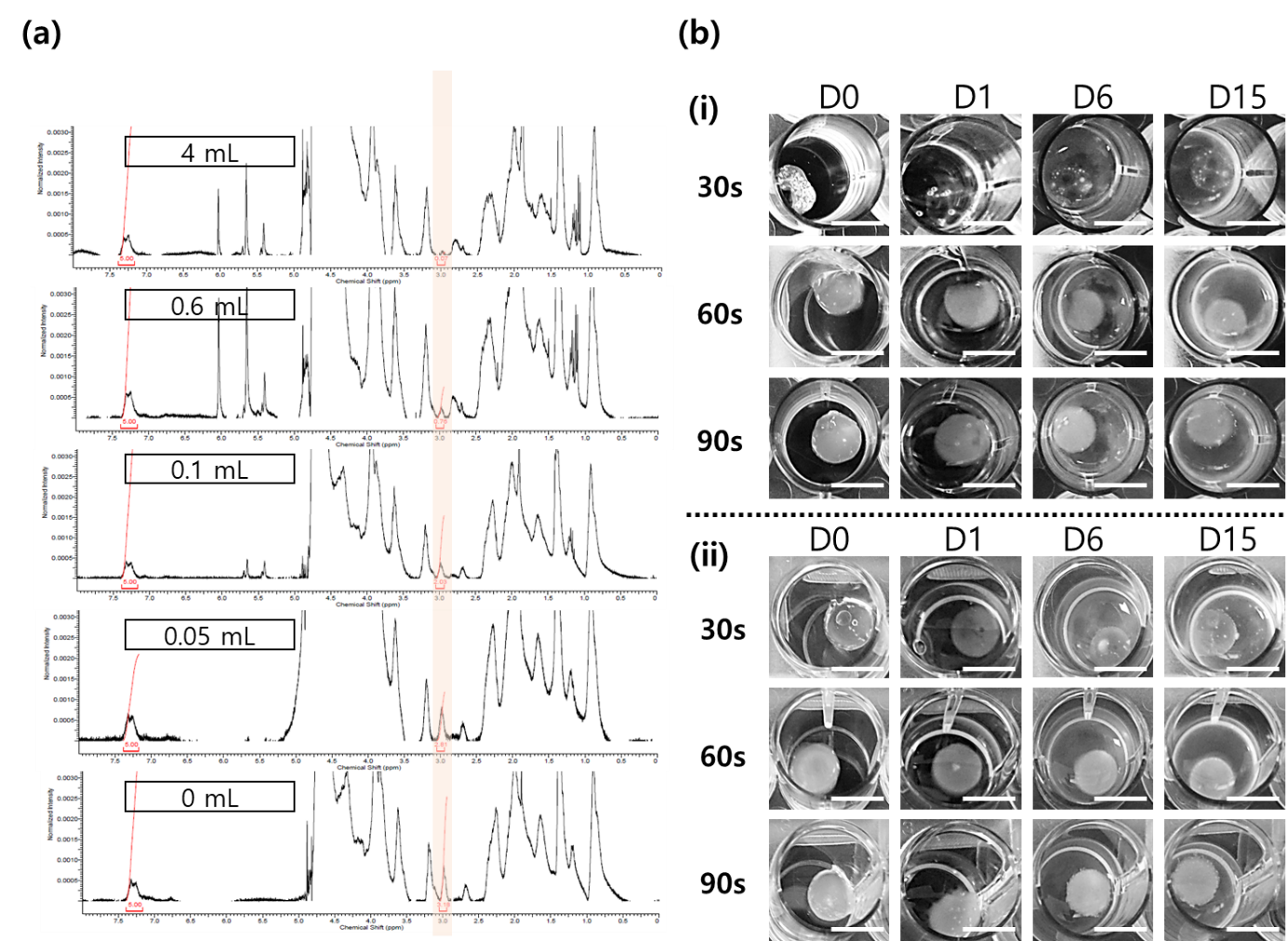


**FIGURE S1**. Characterisation of GelMA (a) ^1^H-NMR analysis. The lysine signal on gelatin was decreased by the addition of methacrylic anhydride, (b) Hydrogel formation depending on GelMA amount ((i) 2.5% and (ii) 5%) and UV treatment time (30–90s), and its integrity in PBS for 15 days. The more stable hydrogel was found by an increase in the GelMA amount and UV treatment time. Scale bars = 1 cm.


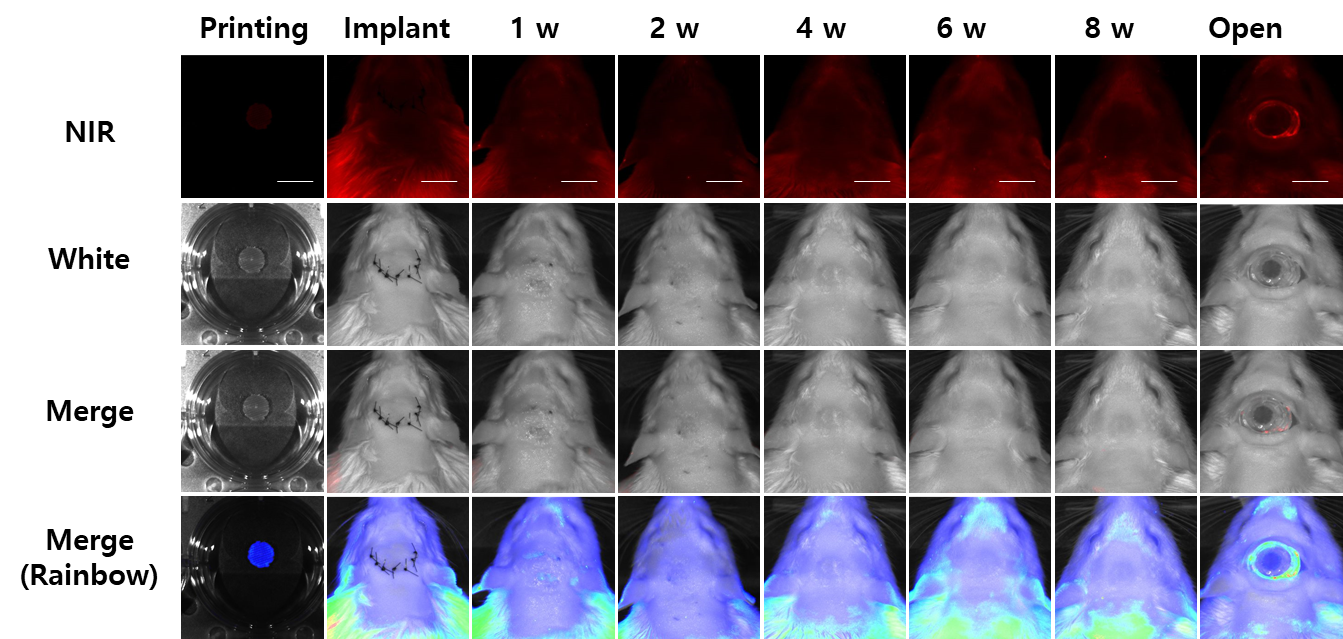


**FIGURE S2**. Control of non-invasive *in vivo* stem cell monitoring as Supplementary information of Figure 5 in the main manuscript. Implantation and observation of the construct (control group: the group implanted the constructs without NIR-MSCs) into the rat calvarial defect site and the non-invasive monitoring for 8 weeks. No NIR fluorescence was emitted from the implants at 700 nm. (The fluorescence at ‘Open’ came from the plastic cap for maintaining opened skin). Scale bars = 1 cm.
